# Smoke Inhalation in a Rural Emergency Setting: A Simulation Session

**DOI:** 10.7759/cureus.784

**Published:** 2016-09-15

**Authors:** Robert Jong, Eugene Krustev, Aron Heroux, Adam Dubrowski

**Affiliations:** 1 Medicine, Memorial University of Newfoundland; 2 Emergency Medicine, Pediatrics, Memorial University of Newfoundland; 3 Marine Institute, Memorial University of Newfoundland

**Keywords:** smoke, inhalation, simulation, rural, emergency medicine

## Abstract

Smoke inhalation-associated lung injuries (SI-ALI) present multiple challenges to the rural emergency department, and they require timely and appropriate management to prevent significant mortality and morbidity. In this report, we outline an adaptable simulation of an SI-ALI patient that is designed for use in a rural emergency department. The aim of this simulation is to better equip clinicians and emergency department staff who may encounter SI-ALI in rural settings. The case is suitable for resident doctors and emergency department staff.

## Introduction

Each year, fire-related burns account for over 450,000 hospitalizations in the United States [1] and over 300,000 deaths per year worldwide [2]. In addition to external burns, 20–30% of burn victims also suffer from smoke inhalation-associated acute lung injury (SI-ALI)  [3]. SI-ALI-induced respiratory complications account for over 35% of burn-related deaths [4]. Because of the high prevalence of SI-ALI amongst burn victims and the morbidity associated with this condition, emergency department physicians, nurses, and trainees need to respond appropriately when managing burn patient airways.

This technical report recreates the arrival of a patient presenting with SI-ALI in a rural emergency department. The objective of this training exercise is to provide clinicians with the following knowledge and skills:

1) Managing smoke inhalation-induced hypoxia

2) Interpreting arterial blood gas results in smoke inhalation

3) Indications for intubation in smoke inhalation and basic post-intubation management.

4) Providing basic life support and advanced airway support including intubation and cricothyrotomy.

This simulation training session is primarily focused on training resident doctors; however, physicians, residents, medical students, nurses, paramedics, and respiratory therapists can, and are encouraged to, participate in the simulation. Furthermore, this scenario is dynamic, allowing for task difficulty to be adjusted in real time to enable the learner to be challenged at a degree appropriate to their skill level [5-6].

## Technical report

All elements, such as the educational context, inputs, processes, and expected products related to the development and implementation of this simulation case, are organized following the Context, Input, Process, Product (CIPP) model [7].

### Context

This simulation could be performed in an emergency department (ED) trauma bay, a medical school, a hospital-based simulation lab, or a hybrid of these settings. Furthermore, the team performing the simulation should have the resident as the main learner, with nurses, physicians, paramedics, or respiratory therapists assisting as needed.

### Inputs

1. Intubating a mannequin with functional upper airway anatomy and cricothyroidotomy landmarks. If a landmarked mannequin is unavailable, appropriate landmarks should be identified by the simulation facilitator.

2. Printed sheets of vitals are to be given or read to the resident upon reaching the next stage of management.

3. Airway equipment:

      •   Oxygen masks

      •   Yankauer suction tip

      •   Laryngoscope blades

      •   Laryngeal mask airway

      •   Bougie

      •   Stylet

      •   Oropharyngeal airway

      •   Endotracheal tubes (5.5–9.0 mm diameter)

      •   Cricothyrotomy intubation kit

      •   IV cannula (Size 14)

      •   Syringe (5 ml)

      •   Bag mask

      •   Normal saline (10 ml)

      •   Scissors

      •   Air tubing

4. Soot moulage to simulate the appearance of a singed oropharynx, tongue, and nares.

5. Emergency department (ED) nurse, if available. If an ED nurse is unavailable, a member of the team can act as one. Alternatively, a trained Standardized Patient (i.e. confederate) could be used.

6. Simulation facilitator(s), who will provide supporting information, ensure adherence to the template, and assess individual and team performance.

7. Simulation driver or technologist (if the mannequin is high fidelity).

8. Optional respiratory therapist to assist with airway equipment set up.

### Process

The ‘code’ leader should be instructed to assign roles in advance. The scenario begins with the leader being told that there was a fire and a patient is being brought into the ED in respiratory distress and with a decreased level of consciousness. The leader will be asked by the facilitator to verbalize what medical issues they anticipate, and, when ready, the team will begin the simulation.

The correct action in this scenario would be for the team to recognize the airway burns and the need for early intubation of the patient. If the leader decides to not intubate this patient, the facilitator should ask them to justify this action, as there are multiple strong indications for intubation in this case. Furthermore, if the leader continues to decide to not intubate, the team should be informed that the patient is now stridorous, or, if a high fidelity mannequin is being used, then the simulation driver should have the patient become stridorous. If this does not prompt the team to intubate, the facilitator of the scenario should act as a staff physician and instruct the team leader to intubate the patient.

The scenario ends when the patient is intubated, appropriate ongoing sedation/analgesia is provided, and plans to transfer, or keep and monitor the patient if transportation is not currently available, are verbalized.

A feedback/debriefing session would then proceed with a review of the team’s performance with the facilitator, preferably initiated through the use of open-ended questioning to evoke useful reflection on the scenario. Specifically, the debriefing should focus on reviewing the learning outcomes and consolidate what they may have learned in the scenario. All feedback should be constructive and respectful. Building on the principles of Advocacy-Inquiry (AI) method of debriefing [8-9], the trainees should also verbalize their thought processes on any mismanaged portions of the scenario and what lead them down that path.

### Product

The expected products, or outcomes, are organized according to the CanMED roles. 

Medical expert: The management of a smoke inhalation case involves clinical decision making, interpreting diagnostic tests, procedural skill proficiency.

Collaborator and communicator: This scenario promotes the use of closed loop communication while working within the healthcare team with a clear definition of roles. If there is any question of how to proceed, advice from team members and allied health staff (ED nurse, respiratory therapist) should be elicited. 

### Case

A 30-year-old male is brought in by emergency medical services (EMS) in respiratory distress after being retrieved from a burning house. The firefighters responded when a neighbour noticed smoke coming from the patient’s home in Goose Bay, NL. An emergency medical team and an ED nurse are immediately available. A respiratory therapist, lab technician, and radiology technician are available on request.

The patient has a decreased level of consciousness (LOC); however, EMS were still able to obtain a brief history from the patient. The patient has been continuously coughing, is short of breath, and his chest and throat hurt. The patient also reports burning eyes and has erythema to his tongue and oropharynx. The patient has scant amounts of soot on his face and neck, as well as in his mouth. His nares are singed. The patient has first-degree burns to his face and neck and arms. There are no other signs of trauma, and the patient does not have a c-spine collar. The patient reports having no allergies, is not currently taking any medications, and does not have any pertinent past medical history. The patient’s last meal was eight hours ago, and his last drink was four hours ago. The patient reports falling asleep on the couch with a glass of scotch and a lit cigar. The patient’s vitals are as follows:

•   Heart rate, 110 bpm

•    Blood pressure, 150/90 mmHg

•    SpO2, 95%

•    Respiratory rate, 40 breaths/min

•    Temperature, 37 °C

After being presented with this information, trainees should initiate and complete the following scenario objectives. 

### Objectives

**Figure 1:**
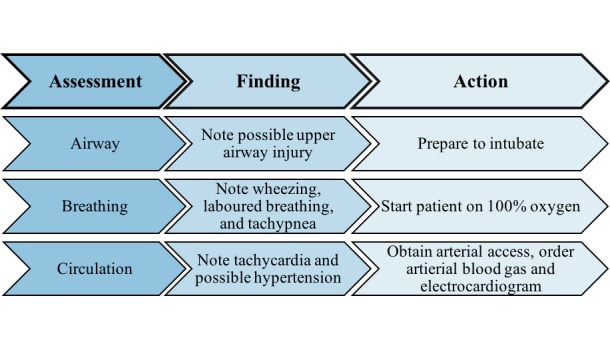
Assessment of airway, breathing, and circulation

Objective 1: Airway, breathing, and circulation (ABCs; Table 1). First, the learners must assess the patient’s ABCs (Figure 1). Upon inspection, the trainees should recognize that singed nares, oropharyngeal erythema, and respiratory distress are indicative of a thermal upper airway injury. Furthermore, the learners should also comment on the fact that the patient’s decreased level of consciousness may impede the patient’s ability to protect his/her airway. Based on this information, the learners should recognize that they must do two things: start the patient on oxygen and prepare to intubate. Before intubation, the learners should obtain venous access. While preparing to intubate, blood work can be ordered, including an arterial blood gas (ABG), as well as an electrocardiograph (ECG). There are two different ABG results that could be provided to the learners if ordered—one if the patient is not on oxygen, and one if the patient is on oxygen (see Appendix). Airway management should not be delayed pending these results.

**Table 1:** The objectives of the case with a snapshot of the vitals expected as the scenario progresses

Objective 1: Airway, Breathing, Circulation		
Vital signs	Expected actions	Cues
HR 100, BP 110/70, temp 37.7, RR 25, O_2_ sat 90%, pH 7.35.	Airway: Note possible upper airway injury and gather all supplies required to intubate. Breathing: Good air entry bilaterally, labored breathing and tachypnea. Start patient on 100% oxygen. Circulation: Note tachycardia and normal blood pressure. Order venous access, arterial blood gas, and electrocardiogram.	Ask the learner to address the ABCs and remember the time sensitivity.
Objective 2: Intubation		
Vital signs	Expected actions	Cues
HR 100, BP 110/70, temp 37.7, RR 22, O_2_ sat 97%	Verbalize why intubation is necessary: decreased level of consciousness and upper airway injury. Discuss necessary medications for intubation. Intubate patient based on Figure 2 for varying skill levels.	Verbally state airway is beginning to swell if learner hesitates on intubation. Verbally prompt learner to explain why intubation is required, and to articulate what medications will be chosen.
Objective 3: Post-Intubation Care		
Vital signs	Expected actions	Cues
HR 100, BP 100/60, temp 37.7, RR 12, O_2_ sat 98%	Discuss post-intubation care and an appropriate analgesic regimen. Contact the intensive care unit and arrange transportation. Order chest X-ray and ...	Verbally prompt patient to explain if and how they would transfer the patient.

Objective 2: Intubation (Table 1). To prepare for intubation the leader should verbalize all materials needed for a difficult airway, including a backup plan if the intubation fails. If the learners have not discussed why intubation is necessary for this patient (decreased level of consciousness and upper airway injury), they should be prompted to do so. Furthermore, the learners should comment on how the patient’s tongue edema might interfere with the intubation. Before beginning the intubation, the trainees need to discuss their approach to intubation in this case and the necessary medications and doses required. Intubation in a patient with a thermal airway injury can be complicated by airway edema and tongue swelling that can potentially obstruct passage of an endotracheal tube. Additionally, loss of resting tone through the use of induction agents in an already vulnerable edematous airway can lead to complete obstruction. 

For these reasons, intubation should ideally be preceded by an 'awake look,' in which topical spray anesthetic is used to advance a laryngoscope with minimal to no IV sedation in order to visualize the cords. At this point, intubation can be performed by passing an endotracheal tube through the visualized cords, or via conventional rapid sequence induction (RSI). A double set-up in which surgical airway anatomy is landmarked and a cricothyroidotomy kit is made available and prepared is also recommended.

**Figure 2:**
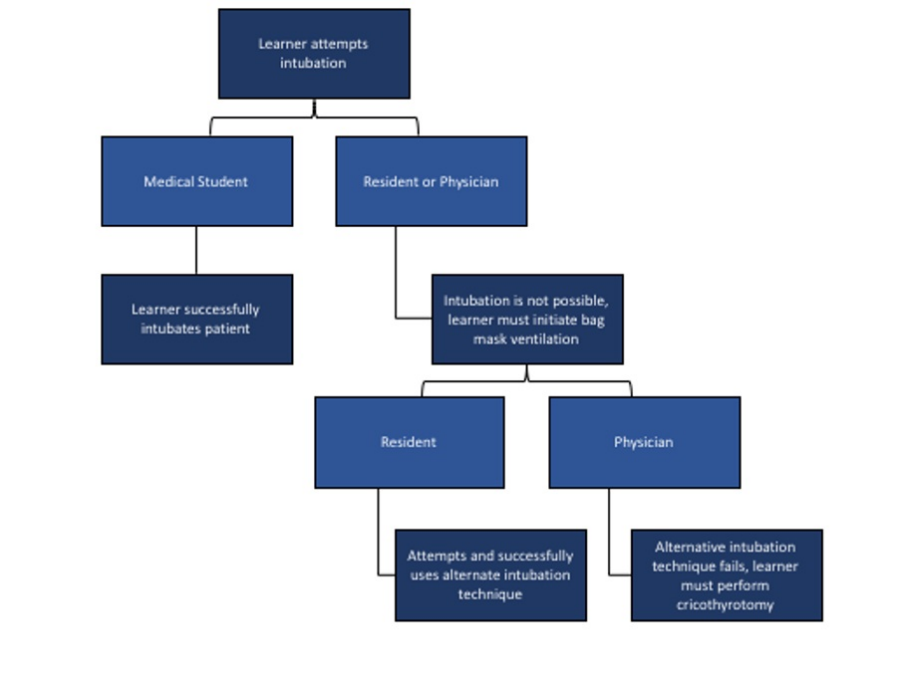
Alternative intubation scenarios for student, resident, and physician learners

Adjusting the difficulty of the scenario. Residents and attending physicians (advanced participants) should describe the steps required for intubation. Resident physicians should then proceed to visualize the airway first using an 'awake look,' and then attempt intubation using their chosen method (see Figure 2). The need for possible surgical airway should be recognized, and if RSI is chosen, an appropriate induction agent and paralytic should be given in rapid succession, followed by attempted intubation with direct or video laryngoscopy (note: there is no contraindication to succinylcholine in this case, as the patient's burn injuries are recent). Appropriate induction medications include, but are not limited to propofol (1–2 mg/kg IV slowly until induction), ketamine (1–4 mg/kg IV push), midazolam (0.1–0.3 mg/kg IV push), and etomidate (0.3 mg/kg IV push). Paralytic agents include succinylcholine (1–1.5 mg/kg IV push) and rocuronium (0.6–1.2 mg/kg IV push). If the leader elects for an awake technique, the patient can be mildly sedated as needed, the patient should be appropriately positioned, and topical spray lidocaine can be used to advance the laryngoscope until a view of the cords is obtained. At this point, if the operator is satisfied with their awake look, they may proceed with induction, paralysis, and intubation while maintaining the view. For resident physicians, the facilitator should allow them to commence intubation. If a high fidelity mannequin is available, airway obstruction should not be activated to allow for a less complicated intubation. A surgical airway should be considered, but is not required for the resident physician participant. In the case of the attending physician, airway obstruction can be activated if a high fidelity mannequin is available. If this is not available, the simulation facilitator should inform the attending physician that complete airway obstruction is present and that intubation will not be successful. The physician leaders should be informed that non-surgical techniques will not work with this patient, and cricothyrotomy should be performed. There are numerous techniques available for performing a bedside cricothyrotomy, and no single technique will be favored. The recommended approach in this paper is an open surgical technique (see Appendix).

Objective 2: Post-intubation care (Table 1). Once a patent airway has been obtained, the trainees begin to discuss post-intubation care and an appropriate analgesic regimen for the patient. The intensive care unit should be contacted, and transportation for the patient should be arranged. The patient’s oxygen saturation should be reassessed, and bonus recognition should be offered to medical students if they recognize that this should be done using arterial blood. Lastly, the trainees should order a chest X-ray (See Appendix) to assess proper device placement as well as occult trauma. 

### Debriefing

At the conclusion of the scenario, a formal debriefing is conducted with the trainees. To establish an environment of psychological safety conducive to learning, the simulation facilitator needs to explain to the learner of the confidential nature of debriefing and also reaffirm their belief in the learner’s intelligence, commitment to doing their best, and desire to improve [8-9].

Post-scenario didactics.After the debriefing session, it is advised to conduct a brief didactic session in which the educators can address any knowledge gaps identified during the scenario and subsequent debrief. This allows the trainees to consolidate new knowledge obtained during the simulation.

## Discussion

The aim of this simulation is to educate learners about proper management of SI-ALI in a rural hospital. Furthermore, this scenario is alterable; therefore, it provides an appropriately challenging learning experience for medical students, residents, or practicing physicians. Specifically, experienced learners will face increased challenges during patient stabilization and intubation, while less experienced learners should gain a basic understanding of managing SI-ALI patients. Regardless of the learner’s level of training, after completing this scenario the learner should have a better understanding of how to manage SI-ALI patients and the technical difficulties that may hinder this process.

Patients presenting with SI-ALI are common in rural health care facilities [10]; therefore, appropriate training for the management of these patients is essential for rural physicians. By participating in this simulation, rural physicians will gain a better understanding of recognizing the presentation of SI-ALI. Furthermore, given that rural health care facilities may have reduced resources for the management of these patients, this scenario is also useful for teaching physicians to work with limited resources and smaller teams.

In addition to the simulation portion of this exercise, learners will also have an opportunity to receive feedback on their performance, as well as reflect on their management of the simulated patient. This debriefing session is essential for explaining to the learner what they did correctly, as well as the areas in which they should aim to improve.

## Conclusions

This simulation is meant to train resident learners how to manage a burn patient presenting with SI-ALI. After completing this scenario, learners should have an improved understanding of how to manage these patients. More specifically, this simulation can be an introductory lesson for beginner students, but should teach more advanced learners and emergency department staff about managing more difficult cases of SI-ALI. By participating in this simulation, health care professionals will be better equipped for responding to patients presenting with SI-ALI in their rural health care facilities.

## Figures and Tables

**Figure 3:**
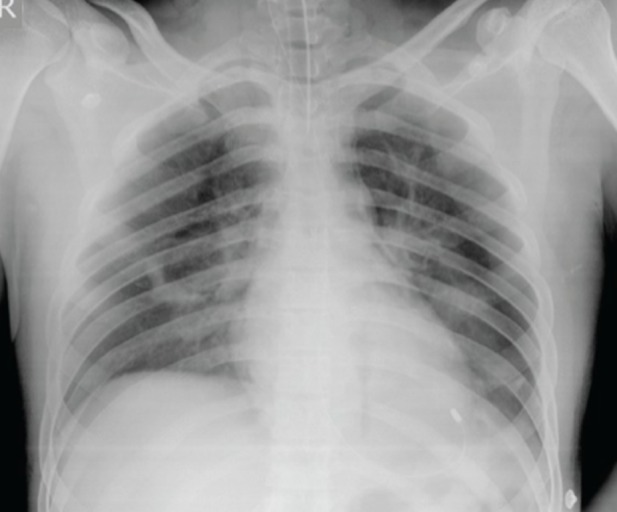
Chest X-ray of intubated patient with smoke inhalation-induced acute lung injury

## Appendices

### Cricothyrotomy

An open surgical technique is described below. Needle techniques can be considered, but they have a much higher failure rate than surgical techniques as described in the literature (the NAP4 audit in the UK describes a 60% failure rate for emergency cannula cricothyrotomy compared to near universal success in the surgical technique).

Ultrasound landmarking should be considered. In one study, only 30% of anesthetists accurately identified the cricothyroid membrane and only 10% marked over the centre point of the membrane [11].

A knife-finger-bougie technique has been described as follows (adapted from Life in the Fastlane: Surgical Cricothyroidotomy [12]):

Indications:

- Can’t intubate, can’t ventilate scenario

Contraindications:

- ability to secure an airway with less invasive means
- airway trauma that renders access via the cricothyroid membrane futile, such as a laryngeal fracture or tracheal transection, in which case a tracheostomy should be performed
- children < 10 years of age, who are prone to laryngeal trauma and in whom a needle cricothyrotomy is generally advised.

Equipment:

- scalpel blade
- artery forceps
- bougie
- size 6-0 endotracheal tube (ETT) or tracheostomy tube

Procedural Steps:

1. Don the mask, visor, gown, and gloves
2. Mark the skin at the location of the cricothyroid membrane
3. Infiltrate this location with local anesthetic with epinephrine
4. Consider sedation if time allows
5. Extend the neck in supine position to make the anatomy more accessible
6. Stabilize the thyroid cartilage with the non-dominant hand
7. Hold the scalpel with the dominant hand and rest it on the patient’s sternum for stability
8. Make a 4-cm vertical incision through the skin over the membrane
9. Palpate the location of the membrane and blunt dissect through subcutaneous tissue until the membrane is readily identified. Ignore bleeding until airway is secure. ETT placement usually has a tamponade effect
10. Make a horizontal incision through the membrane and drag the scalpel blade from one side to the other, then turn the knife through 180 degrees and extend to the other side. The cricothyroid membrane is bound by a cartilaginous cage, so resistance will be felt at the margins of the membrane when the scalpel blade abuts cartilage
11. Dilate with the gloved little finger and palpate tracheal lumen, ideally identifying the cartilage of the posterior wall of the trachea / cricoid ring
12. Pass the bougie alongside the little finger into the trachea
13. Confirm bougie position with the finger, ensuring that it passes through the membrane.
14. The bougie will usually encounter resistance at the carina < 10 cm from the skin and you may feel the tracheal rings as the bougie advances. Do not force the bougie as this may perforate the carina
15. Pass the ETT over the bougie and intubate the trachea. Ensure the ETT balloon is fully deflated and twist the ETT as it passes the skin. Only advance the ETT until the balloon is within the airway and no longer visible (if advanced further, endobronchial intubation is likely)
16. Ensure the ETT is held secure while the bougie is removed and the ETT is connected to the bag-valve-mask** **(BVM)
17. Confirm ETT placement with a combination of end tidal CO2, colorimetry, rise and fall of the chest, fogging of the tube, and subsequent chest X-ray

### Vitals for simulated patient presenting with smoke inhalation-induced acute lung injury

ABG (On O2)

pH - 7.34

PaO2 - 405 mmHg

PaCO2 - 39 mmHg

HCO3 - 22

O2 sat - 71%

HbCO - 28% of total Hb (1-3%)

Meth-Hb - 0.6% (0.1 - 2.0%)

ABG (if not on O2)

pH - 7.29

PaO2 - 200 mmHg

PaCO2 - 39 mmHg

HCO3 - 18

O2 sat - 63%

HbCO - 35% of total Hb (1-3%)

Meth-Hb - 0.8% (0.1 - 2.0%) 

## References

[1] (2016). National Hospital Ambulatory Medical Care Survey: 2011 emergency department summary tables. http://www.cdc.gov/nchs/data/ahcd/nhamcs_emergency/2011_ed_web_tables.pdf.

[2] (2016). World Health Statistics. http://www.who.int/gho/publications/world_health_statistics/EN_WHS08_Full.pdf.

[3] Gorguner M, Akgun M (2010). Acute inhalation injury. Eurasian J Med.

[4] Rex S (2012). Burn injuries. Curr Opin Crit Care.

[5] Guadagnoli M, Morin MP, Dubrowski A (2012). The application of the challenge point framework in medical education. Med Educ.

[6] Parsons M, Murphy J, Alani S, Dubrowski A (2015). Thermal burns and smoke inhalation: a simulation session. Cureus.

[7] Stufflebeam Stufflebeam (2016). CIPP evaluation model checklist. https://www.wmich.edu/sites/default/files/attachments/u350/2014/cippchecklist_mar07.pdf.

[8] Rudolph JW, Simon R, Raemer DB, Eppich WJ (2008). Debriefing as formative assessment: closing performance gaps in medical education. Acad Emerg Med.

[9] Rudolph JW, Simon R, Rivard P, Dufresne RL, Raemer DB (2007). Debriefing with good judgment: combining rigorous feedback with genuine inquiry. Anesthesiol Clin.

[10] Veeravagu A, Yoon BC, Jiang B (2015). National trends in burn and inhalation injury in burn patients: results of analysis of the nationwide inpatient sample database. J Burn Care Res.

[11] Elliot DS, Baker PA, Scott MR, Birch CW, Thompson JM (2010). Accuracy of surface landmark identification for cannula cricothyroidotomy. Anaesthesia.

[12] (2016). Surgical cricothyroidotomy. http://lifeinthefastlane.com/ccc/surgical-cricothyroidotomy/.

